# ‘Here today, gone tomorrow’ – what happened to recurrent brief depression?

**DOI:** 10.1017/S0033291725102912

**Published:** 2025-12-29

**Authors:** Milica M. Nestorovic, David S. Baldwin

**Affiliations:** 1 Clinic of Psychiatry, University Clinical Center of Serbia: Univerzitetski Klinicki Centar Srbije, Belgrade, Serbia; 2 University Department of Psychiatry, Clinical and Experimental Sciences, Faculty of Medicine, University of Southampton, Southampton, UK

**Keywords:** bipolar disorder, major depressive episode, recurrent brief depression, youth mental health

## Abstract

Over twenty years ago, an editorial in this journal called for further studies of the epidemiological and psychopathological characteristics of ‘recurrent brief depression’ in clinical samples in primary and secondary care settings. At that time, relatively little was known about the epidemiology or potential neuropsychobiology of the condition, and no evidence-based treatments had been identified. Two decades have passed, but there have been no substantial developments in understanding in the last ten years. The seeming withdrawal from research into recurrent brief depression is regrettable, given widespread concerns about the burden of depressive symptoms in young people. It seems reasonable to call once again for further investigations in clinical samples, this time with a focus on younger individuals.

Over twenty years ago, in an editorial in this journal, one of us called for further studies of the epidemiological and psychopathological characteristics of ‘recurrent brief depression’ in clinical samples in primary and secondary care settings (Baldwin, 2003). At that time, a category of recurrent brief depressive disorder was included within the 1992 ICD-10 Classification of Disease published by the World Health Organization; and the 1994 DSM-IV manual of the American Psychiatric Association had included this condition within its Appendix B, among disorders worthy of further investigation. The inclusion of recurrent brief depression in the two classificatory schemes probably resulted largely from recognition of the pivotal findings of the longitudinal epidemiological study (the ‘Zurich Study’), led by Angst and Dobler-Mikola (1985), which had shown that it was common (lifetime prevalence approximately 10%), associated with substantial psychiatric comorbidity, often preceded by emotional and behavioral problems during childhood, and with a similar risk of attempted suicide as major depression: but relatively little was known about the epidemiology of recurrent brief depression outside community samples, there had been few investigations of its potential neuropsychobiology, and no evidence-based treatments had been identified. As over two decades have passed, it seems timely to consider any developments in understanding of the condition.

## Current conceptualization of ‘recurrent brief depressive disorder’

In essence, it is characterized by short-lived (less than two weeks) but distressing and often severe depressive symptoms which recur frequently, typically in an ‘irregularly irregular manner’, over many years: in between ‘episodes’, individuals may be troubled by symptoms arising from comorbid conditions, but depressive symptoms are of only mild intensity. Within the 2013 DSM-5 manual of the American Psychiatric Association (DSM-5), the category of ‘recurrent brief depression’ is listed as a type of ‘other specified depressive disorder’ (33 F32.8) which can be used to describe the concurrent presence of depressed mood and at least four other depressive symptoms for at least 2–13 days, occurring at least once per month (though not associated with the menstrual cycle) for at least twelve consecutive months, in an individual whose presentation has never met criteria for any other depressive or bipolar disorder and who does not currently meet active or residual criteria for any psychotic disorder: providing those symptoms are the cause of clinically significant distress or impairment in functioning. By contrast, within the World Health Organization ICD-11 system, ‘recurrent brief depressive disorder’ is a form of ‘other specified recurrent depressive disorder’ (6A7Y) and characterized by the presence of depressive episodes which last less than 2 weeks and occur regularly (e.g. once per month), and which cause significant distress. The DSM-5 conceptualization is therefore stricter than that within the ICD-11, as, for example, someone with a history of a single major depressive episode (even an episode lasting only 15 days) could never be diagnosed as having the pattern of recurrent brief depression.

## Attempts to differentiate it from other mental disorders

Disappointingly, few further attempts have been made to delineate a characteristic psychopathology of recurrent brief depression. Its episodic course and association with suicide risk naturally leads to speculation whether it might represent a *forme fruste* of bipolar disorder. A prospective investigation of daily mood self-reports by over 200 patients with bipolar disorder found that short-lived (2–4 days) depressive episodes were common: using a 2-day threshold, the proportion of patients with depressive episodes was over double that when a 14-day threshold was employed, with no difference between bipolar I and bipolar II subgroups (Bauer et al., 2007). A Norwegian cross-sectional case–control study involving 40 outpatients with recurrent brief depression defined by ICD-10 criteria found that 19 participants had experienced at least three symptoms of hypomania lasting more than one day, this group having a significantly higher proportion of panic disorder but being otherwise indistinguishable from those without hypomanic symptoms (Lövdahl, Andersson, Hynnekleiv, & Malt, 2009): the authors suggested that the presence of hypomanic symptoms should be regarded as a severity marker of recurrent brief depression, rather than an indicator of similarity to bipolar II disorder. The small sample size and cross-sectional nature of the investigation make that suggestion rather speculative, and the same group undertook a subsequent comparison with 21 patients with bipolar II disorder, based on reported somatic and cognitive symptoms, and found some evidence of commonality between patients with recurrent brief depression patients who had experienced hypomanic symptoms and patients with bipolar II disorder, in terms of a small number of symptoms (weakness, easily tired/exhausted, problems concentrating, inability to find words) (Lövdahl, Bøen, Malt, & Malt, 2014, 2015).

Other groups have compared the clinical features of recurrent brief depression with those of other conditions, again in rather small samples. A German group found that short-lived but recurring depressive symptoms were common (70%) among 40 patients with attention-deficit hyperactivity disorder (ADHD) whereas features of ADHD occurred in only a minority (42.5%) of 40 patients with ICD-10 defined recurrent brief depression (Hesslinger, Tebartz van Elst, Mochan, & Ebert, 2003). A US-based group conducting an exploratory statistical analysis of daily mood self-ratings found that individuals with recurrent brief depression (nine participants) differed from individuals with premenstrual dysphoric disorder (15 participants) on the basis of overall variability, approximate entropy (a measure of random variation), and ‘spikiness’ (a measure of incremental variation): the authors suggesting this approach could be used to facilitate differential diagnosis (Pincus, Schmidt, Palladino-Negro, & Rubinow, 2008).

## Any insights into its possible neuropsychobiological basis?

Likewise, there have been no major advances in understanding of the etiology of recurrent brief depression. A Spanish polysomnographic investigation which included patients with DSM-III-R defined borderline personality disorder or major depressive disorder, recurrent brief depression defined by criteria similar to those in ICD-10, and healthy controls (each of the four groups comprising 20 participants) found no differences between patients with recurrent brief depression and healthy controls in total sleep time or percentage of wakefulness, although all patient groups had longer sleep latency than healthy controls (De la Fuente et al., 2004). A Norwegian exploratory functional magnetic resonance imaging study involving a working memory paradigm (the *n*-back working memory task) in 22 female patients with ICD-10-defined recurrent brief depression (divided equally between those with or without a history of brief hypomanic episodes) found some evidence of poor task modulation in the left dorsolateral prefrontal region (in both subgroups), together with a lack of deactivation within the right insula in the patient sub-group with a history of brief hypomanic episodes: the authors speculating that this reflects a need for greater cognitive control when those individuals perform the cognitive task (Korsnes et al., 2013). This group also compared 46 unmedicated patients with recurrent brief depression and 24 matched controls on a range of cognitive tasks (assessing working memory, attention, verbal/visual memory, psychomotor speed and executive function) and found evidence of impairment in cognitive domains other than verbal learning and nonsematic verbal fluency, these differences being independent of variables relating to severity, duration, and periodicity of depressive symptoms (Andersson, Lövdahl, & Malt, 2010).

Case reports have suggested an association of recurrent brief depression with agenesis of the corpus callosum, and with Fabry disease (an X-linked deficiency leading to accumulation of glycosphingolipids), but these may be only chance associations. As with major depression, it has been suggested that recurrent brief depression may be associated with persistent immune activation: in a case-controlled study, patients (n = 135) with chronic hepatitis C infection (and without a history of hepatitis B or human immunodeficiency virus infection, interferon-alpha treatment, malignancy, or drug or alcohol abuse) had an elevated cross-sectional prevalence (15.5%) of recurrent brief depression when compared to controls (n = 540) without hepatitis (6.3%) (Carta et al., 2012). The autoimmune condition celiac disease, characterized by gluten enteropathy and often by wider systemic manifestations, has also been found to be associated with a high prevalence of recurrent brief depression (36.1% of 36 adult patients) when compared to healthy controls (6.9% of 144 individuals) (Carta, Hardoy, Usai, Carpiniello, & Angst, 2003).

Investigations of personality characteristics have not provided pivotal insights into recurrent brief depression. A cross-sectional survey in a single United Kingdom general practice (which found that 40% of patients with ‘anxiety’ and 21% of patients with an allergy experience DSM-IV-defined recurrent brief depressive episodes), indicated that – as might be anticipated - those with such episodes (n = 45) had significantly higher levels of state–trait anxiety and neuroticism and lower levels of extraversion than those individuals without depression, or when compared to normative values for the population (Williams, Richards, Ameen, & Davies, 2007). An investigation of temperament and character variables suggested some similarities between 40 patients with recurrent brief depression (ICD-10-defined) and 21 patients with bipolar II disorder: when compared to 19 age- and sex-matched healthy controls, both patient groups showed evidence of increased harm avoidance, lowered self-directedness, and increased self-transcendence: however, most differences from healthy controls disappeared after controlling for comorbid panic disorder (Lövdahl, Bøen, Falkum, Hynnekleiv, & Malt, 2010).

## Is there now an established evidence-based treatment?

Unfortunately, no specific pharmacological or psychotherapeutic treatment has been identified. Only one double-blind, randomised, placebo-controlled study (n = 35) has been published in the last two decades, in which we found no significant advantage over placebo for either the tricyclic antidepressant imipramine or the reversible inhibitor of monoamine oxidase A moclobemide in reducing the severity, duration or frequency of depressive episodes over four months of double-blind treatment (Baldwin, Green, & Montgomery, 2014). The lack of efficacy of these antidepressants mirrors a previous finding of the lack of efficacy of the selective serotonin reuptake inhibitor fluoxetine in reducing depressive episodes and nonfatal self-harm among patients with a history of repeated suicide attempts. Case reports have described beneficial effects of the selective serotonin reuptake inhibitor paroxetine, the dopamine D2 receptor and serotonin (5-hydroxytryptamine) 5-HT2 receptor antagonist olanzapine, and the mood-stabilizing drugs lamotrigine and sodium valproate, but randomized placebo-controlled trials with these or other compounds are needed before specific treatments can be recommended. While certain psychotherapeutic interventions can be beneficial in reducing affective instability and emotional dysregulation across a range of mental disorders, no specific psychotherapeutic treatment for recurrent brief depression has been identified.

## But is there a relationship to the increasing prevalence of depression in young people?

It is worth considering whether the experience of recurrent brief depression is an important contributor to reports of depression in young people. The modern description of ‘recurrent brief depression’ is derived from the findings of the longitudinal epidemiological Zurich study, with its careful serial interviews of an enriched population of community-dwelling individuals who were aged 18 years at inception into the cohort (Angst & Dobler-Mikola, 1985). Findings from other study samples also attest to the high prevalence and early onset of the condition: for example, a mixed urban–rural community survey in Sardinia, Italy (1040 participants) found a 13.8% ‘lifetime prevalence’ of recurrent brief depression among 1040 participants aged 18–24 years (Carta et al., 2003).

A systematic review of ‘subthreshold depression’ in adolescence (age 10–19 years), which included 27 cross-sectional or longitudinal studies, in which depressive symptoms either fell below the requisite number of symptoms and/or the 2-week duration of symptoms for major depressive episodes (many studies required a duration of only 1 week), drew attention to the rising prevalence of depressive symptoms in young people, and to the need for consensus on the definition of subthreshold depression, to facilitate further research (Bertha & Balázs, 2013). An analysis of 36 national surveys in England, Scotland, and Wales during the period 1995–2014, involving a total of 140,830 participants, found a ‘striking increase’ in the reported prevalence of long-standing mental health conditions among children and young people (participants aged less than 25 years) (Pitchforth et al., 2019). Furthermore, a recent comprehensive overview stated that depression, ‘no matter how it is defined’, is becoming more common in later adolescence and early adulthood than in previous years, especially in females (Thapar, Eyre, Patel, & Brent, 2022), who are more likely to have a course of illness characterized by chronicity, relapses, or recurrences (Tagliaferri et al., 2025). However, the specific contribution of short-lived (i.e. less than two weeks), but frequently recurring depressive symptoms to the rising burden of depression in young people has not been adequately evaluated.

## A continuing need

The call within the 2003 Editorial for further epidemiological and psychopathological studies of recurrent brief depression in patient groups does not appear to have been heard widely: only a few studies have been published, by even fewer research groups, most investigations being in small samples, the vast majority being in the first rather than the second decade. It seems reasonable to question why no major contributions to understanding of recurrent brief depression have been made in the last decade. Skeptical clinicians often distrust ‘new’ diagnostic entities and could feel that the boundaries between it and other affective disorders are insufficiently defined. Disappointing findings from randomized placebo-controlled treatment studies may have disincentivized biotechnology companies previously interested in novel pharmaceutical targets, and clinical researchers could have been discouraged from further exploration of its possible neuropsychobiological basis. The lack of an established treatment for the condition represents a significant block to its recognition by hard-pressed health professionals working in the hurly-burly of routine clinical practice. But this withdrawal from research into the psychopathology, etiology, and treatment of recurrent brief depression is particularly regrettable, given widespread concerns about the burden of depressive symptoms in young people. It seems reasonable to call once again for further investigations of the epidemiology, neuropsychobiology, and treatment of recurrent brief depression in clinical samples, with consideration of predictors of the course of illness, evolution of comorbidity, and suicide risk, but this time with a sharpened focus on younger individuals.
